# Histological changes in placental rat apoptosis via FasL and cytochrome *c* by the nano-herbal *Zanthoxylum acanthopodium*

**DOI:** 10.1016/j.sjbs.2021.02.047

**Published:** 2021-02-18

**Authors:** Putri Cahaya Situmorang, Syafruddin Ilyas, Salomo Hutahaean, Rosidah Rosidah

**Affiliations:** a Department of Biology, Faculty of Mathematics and Natural Sciences, Universitas Sumatera Utara, Medan, Indonesia; b Faculty of Pharmacy, Department of Pharmacology, Universitas Sumatera Utara, Medan, Indonesia

**Keywords:** Apoptosis, Cytochrome *c*, FasL, Hypertension, *Zanthoxylum*, PE, Preeclampsia, EVOO, Extra Virgin Olive Oil, MDA, Malondialdehyde, HSP-70, Head Shock Protein-70, T1, Hypertension rats given EVOO, T2, Hypertension rats given Nano herbal Andaliman, T3, Hypertension rats given EVOO and Nano herbal Andaliman, C, control, ZA, *Zanthoxylum acanthopodium*

## Abstract

*Zanthoxylum acanthopodium* has several biological activities, such as antimicrobial, anti-inflammatory, and antioxidant properties. This strong antioxidant herb can be used as a drug for hypertension. FasL and cytochrome *c* expression play roles in the apoptotic pathway in the placenta. This study focused on the histological change in apoptosis via cytochrome *c* and Fas ligand expression by investigating whether *Zanthoxylum acanthopodium* (ZA) fruits affect apoptosis. The present study consisted of five treatments: Normal pregnant rats (C), Hypertension rats (C + ), hypertension rats + extra virgin olive oil (EVOO) (T1), Hypertension rats + ZA (T2), and hypertension rats + EVOO + ZA (T3). Hypertension was induced in rats by injecting 3 mlml of 6% NaCl. Nanoherbal of ZA (100 mg/kg BW) and EVOO (1 ml) were given on the 13th–19th days of pregnancy. Pregnant rats were dissected on the 20th day of pregnancy by cervical dislocation. ELISA assays were performed for the analysis of HSP-70 expression. Immunohistochemistry and TUNEL assays were used to analyse the histological changes in placental tissue. The results showed that cytochrome *c* and FasL protein exposure levels in the labyrinth, basal, and yolk sac zones were increased during hypertensive pregnancy (P < 0.0001) in rats. The administration of nanoherbal of ZA decreased the expression of cytochrome *c* and FasL. A significant difference was found in the combination of nanoherbal of ZA and EVOO.

## Highlights

• The administration of nanoherbal andaliman reduced apoptosis via cytochrome *c* and FasL.
• EVOO reduces apoptosis via cytochrome *c* and FasL better than andaliman.
• Combination Nano herbal andaliman and EVOO reduce reduced apoptosis via cytochrome *c* and FasL on placental histology of hypertension rats.
• Combination Nano herbal andaliman and EVOO reduce MDA levels in hypertension rats.
• Nano herbal andaliman and combined with EVOO increase HSP-70 expression in hypertension rats.

## 1 Introduction

Fas ligand (FasL) and cytochrome *c* expressions play roles in the apoptotic pathway in placental hypertension or preeclampsia (PE). Decreased FasL expression, increased apoptosis, and syncytial node formation could be involved in the pathophysiological mechanisms of hypertension and PE (Tomas et al., 2011). FasL and its receptor play an important role in the regulation of the immune response. FasL is expressed by activated T cells, cytotoxic T lymphocytes, Sertoli cells of the testis, and corneal epithelium and endothelium (Tomas et al., 2011, Hasan et al., 2020). The major function of Fas/FasL interaction and Fas activation is the induction of cell apoptosis FasL expression is generally less in villus (Hasan et al., 2020). Cytochrome *c* can be activated by an adapter protein, namely, APAF-1, in the intrinsic pathway of the mitochondria. An active cytochrome *c* provides strong immunoreactivity in the preeclamptic placenta (Can et al., 2014). The induction of a potential substance can play a role in releasing cytochrome *c*, dividing PolyADP-ribose polymerase (PARP), and activating caspase 3. This process showed condensed chromatin and positive staining by TUNEL assay and they associated with an increase in the number of nuclei (Vo et al., 2020). A tumour in placenta is an apoptotic pathway caused by the cytochrome *c* in the rat placenta, and hypoxia-reoxygenation (in vitro) stimulates apoptosis in placental tissues. This remarkable increase in the release of cytochrome *c* from the mitochondria is associated with intensive immunolabelling for active caspase 3 in endothelial syncytiotrophoblast cells and foetuses (Mohan et al., 2016, Lu and Sferruzzi-Perri, 2020).

*Zanthoxylum acanthopodium* (ZA) local name as andaliman is an endemic plant from Sumatera Utara province, Indonesia (Wijaya et al., 2019). ZA fruits is used by the community as a food ingredient because of its orange-like fragrance. The fruit of this plant has several biological activities and antimicrobial, anti-inflammatory, and antioxidant properties (Wijaya et al., 2019). A fraction of alkaloid ZA fruit has strong antioxidant activity (Rosidah et al., 2018). The strong antioxidant activity can be used as a drug for preeclampsia (PE) because antioxidant factors participate in the etiology of PE. In Indonesia, PE is the second hazardous pregnancy disease after bleeding (Situmorang et al., 2019a). The levels of antioxidants of ZA such as essential oil, coenzyme 10, certain vitamins, and lycopene are reduced during preeclamptic pregnancy (Haram et al., 2019). Excessive increase in the oxidative agents of ischemia–reperfusion in the placenta can affect the normal antioxidant activity, thereby causing PE pathogenesis (Situmorang et al., 2019a). ZA combined with *Extra Virgin Olive Oil* (EVOO) can reduce systolic blood pressure and change MDA and HSP-70 in preeclamptic rats (Situmorang et al., 2019a, Situmorang et al., 2019b). Moreover, ZA affects the placental and renal histology of pre-eclamptic rats (Situmorang et al., 2019a, Situmorang et al., 2019b). Pure of ZA seed extract also has a powerful effect in inhibiting the proliferation of MCF-7 cell lines (Arsita et al., 2019).

In this study, we focused on the description of cytochrome *c* and FasL expressions by investigating whether effect *Zanthoxylum acanthopodium* fruits in histology placental. In the setting of our experiments, we've also given *Extra Virgin Olive Oil* (EVOO) to 1 group for the rats PE (in vivo study) as pure antioxidants and the comparison with ZA. The antioxidants of EVOO is important from health, biological, and sensory points of view. It has been proven by many researchers. Lipophilic, phenols, and hydrophilic (include a large variety of compounds) represent the main antioxidants of EVOO (Servili et al., 2014). EVOO can protect against induced cell injury through oxidative stress, inflammatory, and apoptosis in rats model (Elgebaly et al., 2018, Cárdeno et al., 2014).

## 2 Materials and methods

### 2.1 Preparation of nanoherbal Zanthoxylum acanthopodium (ZA)

*Zanthoxylum acanthopodium* fruit was from Berastagi, Sumatera Utara, Indonesia. Nanoherbal of ZA was produced using a *High-energy milling* tool (HEM-3D, HCl 2 M activator solution Tokyo, Japan) for 2 h in LIPI Jakarta. Pure EVOO was purchased from supermarkets in Medan (Bertolli, Italia; Sertifikat: IFS–BRC). EVOO dose calculation was based on previous studies (Ilyas et al., 2018, Situmorang et al., 2019a).

### 2.2 Experimental animals

The current research was conducted at the Biology Laboratory of the University of Sumatera Utara, the Pathology and Anatomy Laboratory of the University of Sumatera Utara, and the Indonesian Institute of Education and Research, Jakarta, Indonesia from October 2017 until June 2020. A total of 50 Wistar or 25 pairs (180–250 g) *Rattus norvegius* from the Animal cage of the University of Sumatera Utara, Indonesia were used as experimental animals. The study with a posttest only control group design. The rats were kept in the standardized animal cages with stable temperature and relative humidity. The rats were given standardized rat pellets and water compliance with the Biology Deparment of University of Sumatera Utara laboratory protocol. The research was approved by the Health Research Ethics Committee of USU Medan (Ethical Clearance: No. 010/KEPH-FMIPA/2020).

### 2.3 Rats models of hypertension

Male and Female rats were acclimatized to laboratory condition for 2 week before the study and the rats were given standardized rat pellets and abundant water. The female rat mated in one cage with male rat overnight in the estrous cycle. The 0th pregnancy was determined by finding a vaginal plug. The preeclamptic model rats were produced by injecting 3 ml of 6% NaCl subcutaneously on the 6th–12th day of pregnancy (Situmorang and Ilyas, 2018, Situmorang et al., 2020, Situmorang et al., 2019c). On day 13 of pregnancy, rats were randomized into five groups (n = 5) and the blood pressure and proteunaria were calculated. The present study consisted of five treatments: untreated pregnant rats (C), hypertension rats (C+); hypertension rats + EVOO (T1); hypertension rats + ZA (T2), and hypertension rats + EVOO + ZA (T3).

### 2.4 Measurement of blood pressure

Measurement of systolic and diastolic blood pressure using a volume pressure recording sensor by CODA non-invasive system (Kent Scientific Corporation) on day 5 of pregnancy (before 6% NaCl injection), day 13 of pregnancy (After 6% NaCl injection/before administration of nanoherbal ZA or EVOO) and day 20 of pregnancy (before dissected) through the base of the tail.

### 2.5 Measurement of Malondialdehyde (MDA)

The rats were dissected on 20 day of pregnancy by cervical dislocation. Blood was collected via the hearts in vacuum tubes. The Blood rats was centrifuged at 3.000 rpm for 20 min to get the serum. The procedure was carried out by following the instruction by a manufacturer on MDA measurement and the samples read with a spectrophotometer (UV2400 Spectrophotometer: Zhejiang, China) with a wavelength of 530 nm (Situmorang et al., 2019a).

### 2.6 Measurement of heat Shock Protein-70 (HSP70)

The Blood was collected from the rats hearts in vacuum tubes, centrifuged at 3000 rotations per min for 15 min using the DT5-6A (2) low-speed centrifuge (Tianjin, China). The procedure was carried out by following the instruction by a manufacturer on HSP70 measurement (catalog no. MBS725078, 2–8 °C, 1:4 dilutions) and the samples read with Well Reader-Elisa Reader R-Biopharm (Germany) with a wavelength of 450 nm (Situmorang et al., 2019a).

### 2.7 Immunohistochemistry

The placental tissue was added to the formalin, and samples are cut with a thickness of 4 μm to make the paraffin block. Slides were used for immunohistochemical staining. FasL detection used Fas-L mouse monoclonal antibodies (NOK-1):sc-19681 (dilution 1:50 with PBS, Santa Crus Biotechnology, Santa Cruz, CA, USA), and cytochrome *c* detection used a monoclonal mouse anti-cytochrome *C* antibody (ready to use) 7H8.2C12 (Medaysis Enable Innovation Company), formulation in PBS pH 7.4, containing BSA and ≤0.09% sodium aide (NaN_3_). Placental tissue was rehydrated and incubated (5 min) in 3% hydrogen peroxide and distilled water. For pre-treatment, the tissue was heated (10 min) in citrate buffer at pH 6.0 and 350 W. After washing with PBS, the tissue was incubated (15 min and 120 min) with FasL and cytochrome *C* antibodies, respectively, at 37 °C then washed again with PBS before applying avidin–biotin peroxidase. 3,3-Diaminobenzidine (DAB) hydrochloride was used for chromogenic visualisation reaction and then stained with haematoxylin Mayer (30 s). The slides are inserted to the ethanol series and finally through xylene and was covered with a glass cover with Canadian balm (Merck, Darmstadt, Germany).

### 2.8 TUNEL assay

The placental tissue in the slide was immersed in fresh xylene for 5 min. The paraffin-embedded part was stained with TUNEL techniques with detection kit (Promega, Cat # G7130; USA). The slides were rehydrated with multilevel ethanol (3 min) and washed with NaCl 0.85% and PBS for 5 min. After rehydration, incubation was conducted for 15 min at room temperature with proteinase K (20 μg/ml). The final labelling reaction was carried out by adding the reaction mixture of rTdT to the slides in a humid room (37 °C for 1 h). The reaction of rTdT enzyme was ended by immersing the slide in a buffer at room temperature (15 min). The slides were washed 5 min with PBS. The endogenous peroxidase was blocked by 0.3% hydrogen peroxide to PBS (30 min). A streptavidin-HRP solution was added to the tissue and incubated at room temperature (30 min). A chromogenic substrate DAB was added to the slide. All slides were dehydrated with graded ethanol (3 min) and cleaned in 100% xylene for 5 min each with three times.

### 2.9 Statistics

The research data were analyzed using one-way ANOVA in Sigmaplot software. Data were analyzed with averages and standard deviations. Asterisks indicate the level of statistical significance (*P < 0.05, **P < 0.01, ***P < 0.001 ****P < 0.0001, ns = P > 0.05).

## 3 Results

### 3.1 Blood pressure, MDA, and HSP70 after administration of nano-herbal ZA and EVOO

Table 1 shows the systolic and diastolic blood pressure and MDA and HSP70 levels in pregnant rats as parameters for hypertensive rats. There was an insignificant difference (P > 0.05) on day 5 before the injection of 3 mlof 6% NaCl, but after the injection of 3 ml of 6% NaCl (day 13), there was a significant difference between groups C− and C+ (P < 0.01, F = 0.0098). Administration of 6% NaCl can increase systolic and diastolic blood pressure, although the increase is not significant for diastolic blood pressure. On day 20 (before dissection/after herbal administration), there were significant differences in systolic blood pressure in groups C- and C+ (P < 0.01, F = 0.0076) and T1 (P < 0.05, F = 0.043), T2 (P < 0.05, F = 0.040), and T3 (P < 0.05, F = 0.043) compared with group C+. However, the differences in diastolic blood pressure were not significant. Table 1 also shows that MDA levels in C+ were highest (P < 0.01, F = 0.0085) and HSP70 levels were low (P < 0.05, F = 0.046). Blood pressure, MDA, and HSP-70 can be used as markers of preeclampsia or hypertension.

**Table 1:** The blood pressures, MDA and HSP70 on preeclamptic rats.

Groups	Systolic Blood Pressures (mm/Hg)			Diastolic Blood Pressures (mm/Hg)			MDA	HSP70
	Days							
	5	13	20	5	13	20		
C-	120.3 ± 4.08	120.3 ± 4.01	120.1 ± 3.98	80.1 ± 3.28	80,1 ± 2.98	80.0 ± 3.21	0.36 ± 0.05	38.9 ± 3.61
C+	120.6 ± 5.01 ^ns^	140.2 ± 4.08**	142.7 ± 4.81**	79.2 ± 5.21 ^ns^	90.2 ± 5.21 ^ns^	80.8 ± 4.29 ^ns^	0.61 ± 0.06**	23.8 ± 3.85*
T1	118.6 ± 4.09 ^ns^	138.6 ± 9.76 ^ns^	121.6 ± 4.79*	79.8 ± 4.49 ^ns^	88.8 ± 4.49 ^ns^	82.8 ± 4.39 ^ns^	0.42 ± 0.08 ^ns^	36.1 ± 4.76*
T2	119.6 ± 5.08 ^ns^	141.6 ± 8.21 ^ns^	127.9 ± 5.11*	80.3 ± 5.09 ^ns^	87.3 ± 5.09 ^ns^	83.8 ± 8.91 ^ns^	0.47 ± 0.07 ^ns^	27.2 ± 4.66 ^ns^
T3	119.4 ± 4.97 ^ns^	129.4 ± 5.11*	121.8 ± 3.88*	81.2 ± 5.98 ^ns^	89.2 ± 5.98 ^ns^	80.9 ± 7.28 ^ns^	0.40 ± 0.06 ^ns^	35.9 ± 5.21*

C-: untreated pregnant rats, C+: Hypertension rats, T1; Hypertension rats + EVOO, T2: Hypertension rats + andaliman, T3: Hypertension rats + EVOO + andaliman. (*P < 0.05, **P < 0.01, ***P < 0.001, ^ns^ = P > 0.05/ Not significant).

(*P < 0.05, **P < 0.01, ns = P > 0.05).

### 3.2 Number of foetal births in the treatments

**Fig. 1:**
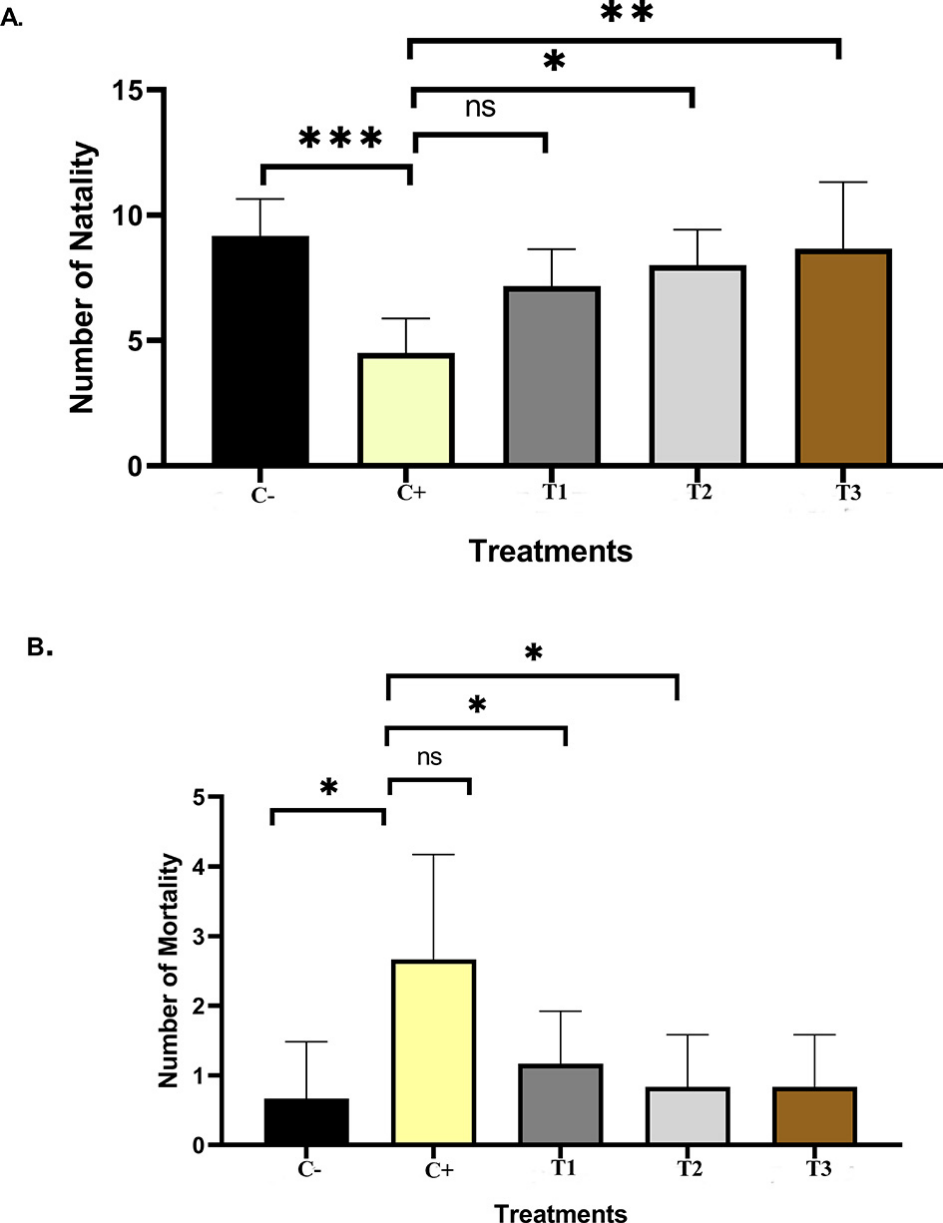
A number of Fetall Birth in the treatments. A. Natality, B. Mortality C-: untreated pregnant rats, C+: Hypertension rats, T1; Hypertension rats + EVOO, T2: Hypertension rats + ZA, T3: Hypertension rats + EVOO + ZA (*P < 0.05, **P < 0.01, ***P < 0.001 ****P < 0.0001, ns = P > 0.05).

Fig. 1A shows the number of significant rat foetal births with a value of F = 0.0009 (P < 0.001) in foetal natality. In the foetal natality treatment group, significant differences were observed in the T2 (F = 0.030, P < 0.05) and T3 (F = 0.0151, P < 0.01) groups compared with the C+ group. However, the T1 group was insignificant (F = 0.093, P > 0.05). Fig. 1B also shows the number of significant foetal deaths in rats, with a value of F = 0.0081 (P < 0.01). Significant differences were observed in the control (F = 0.011, P < 0.05), T2 (F = 0.022, P < 0.05), and T3 (F = 0.022, P < 0.05) groups compared with the C+ group. No significant difference was found in T1 compared with the C+ group (F = 0.0826, P > 0.05). Based on the graph in Fig. 1, hypertension can affect the number of foetal births.

### 3.3 Histological changes in placental tissue apoptosis via cytochrome *c* and FasL in normal pregnant rats

**Fig. 2:**
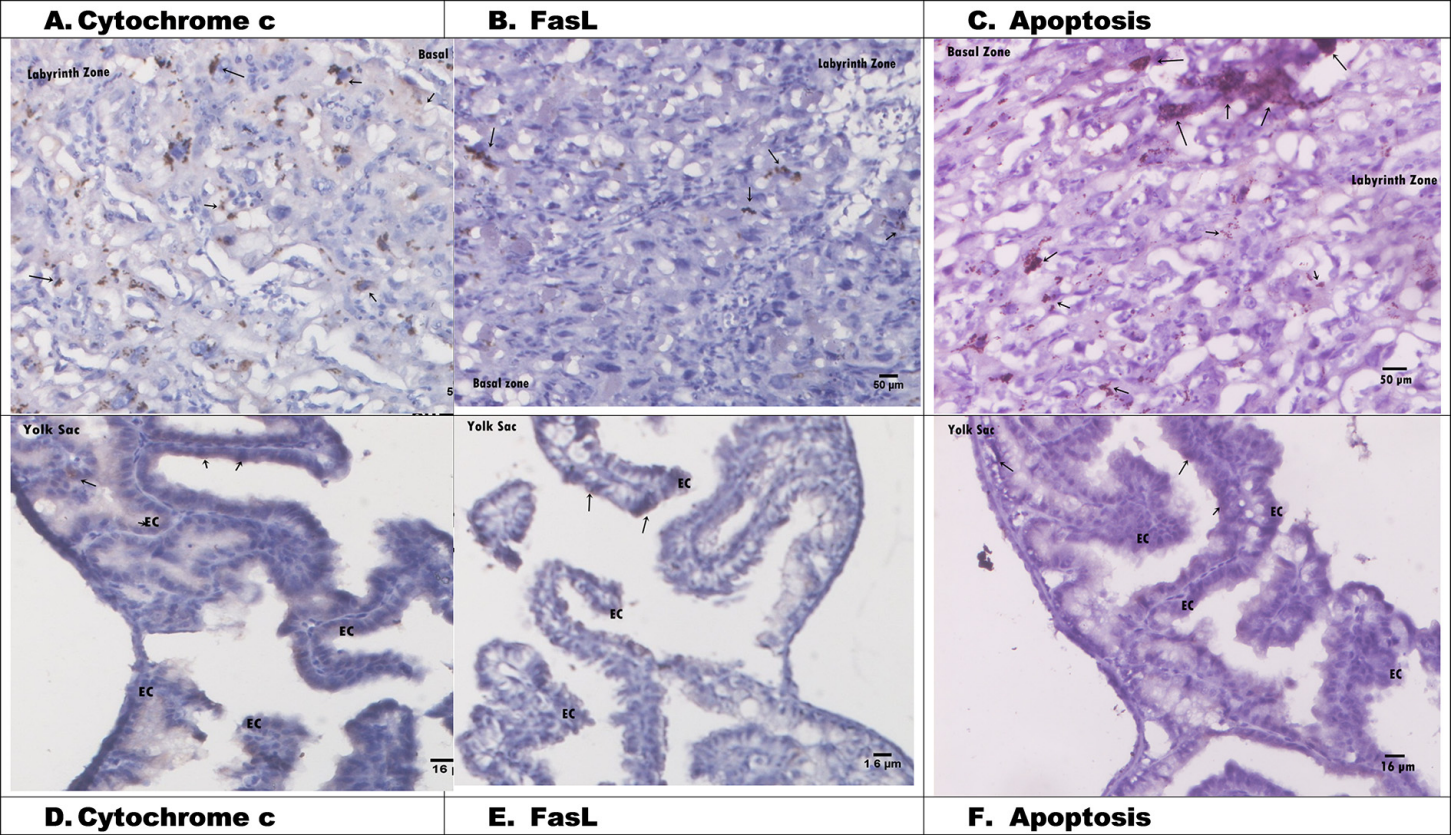
Histology of rats placental without treatments (C-l: A. Cytochrome *c* (50 µm), B. FasL (50 µm), C. Apoptosis (50 µm). D. Cytochrome *c* (16 µm), E. FasL (16 µm), F. Apoptosis (16 µm). EC: Epithelial Cells, FV: Fetal vessels.

Fig. 2 shows the placental histology in the control group. Cytochrome *c* expression was indicated by dark brown stains (Fig. 2A). In the labyrinth zone, spongiotrophoblasts were present just above the giant trophoblast cells located at the materno-foetal placental interface. Cytochrome *c* expression was also shown in epithelial cells (ECs) in the yolk sac of the placenta (Fig. 2D). FasL antibody expression in the control group was less than cytochrome *c* expression (Fig. 2B). FasL was rarely observed in the nuclei of giant trophoblast cells and in certain intracellular vesicles. Even epithelial uterine and columnar trophoblast cells (CTCs) were negative. FasL expression was clearly visible in the labyrinth and basal zones compared with the yolk sac (Fig. 2B and E). The yolk sac consisted of epithelial and mesodermal cells, which were divided into visceral and parietal parts. Based on the placental histology in Fig. 2, the expression levels of cytochrome *c*, FasL, and apoptotic cells were safe and normal in the control group.

### 3.4 Histological changes in placental tissue apoptosis via cytochrome *c* and FasL in hypertensive pregnant rats

**Fig. 3:**
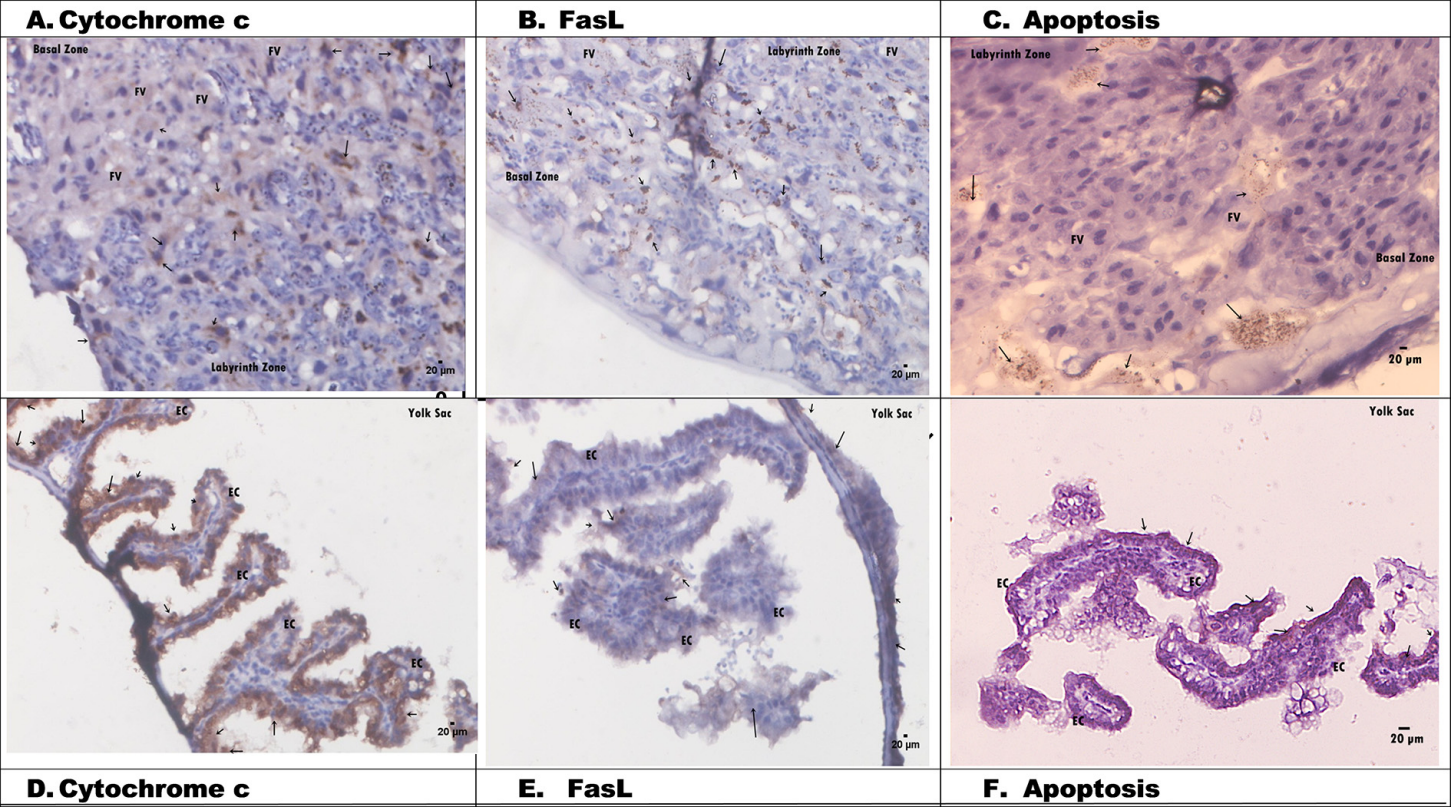
Histology of hypertension rats placental (C+): A. Cytochrome *c* (20 µm), B. FasL (20 µm), C. Apoptosis (20 µm). D. Cytochrome *c* (20 µm), E. FasL (20 µm), F. Apoptosis (20 µm). EC: Epithelial Cells, FV: Fetal vessels.

Fig. 3 shows the placental histology after injection of 3 mlml of 6% NaCl. Cytochrome *c* and FasL expression in the C+ group were indicated by brown in the labyrinth and basal zones. Brown-coloured nuclei were characterized by nuclear fragmentation, cytoplasmic dehydration, and condensation of chromatin but preservation of membrane cell integration. Cytochrome *c* expression in C+ often occurred in cells near the foetal vessel (FV) (Fig. 3A and D). Apoptosis was also observed in the labyrinth zone, as shown by placental histology (Fig. 3C). Based on the placental histology of the hypertension group, apoptosis via cytochrome *c* and FasL increased during pregnancy.

### 3.5 Histological changes of placental tissue on apoptosis via cytochrome *c* and FasL in hypertensive pregnant rats after extra virgin olive oil (EVOO) administration

**Fig. 4:**
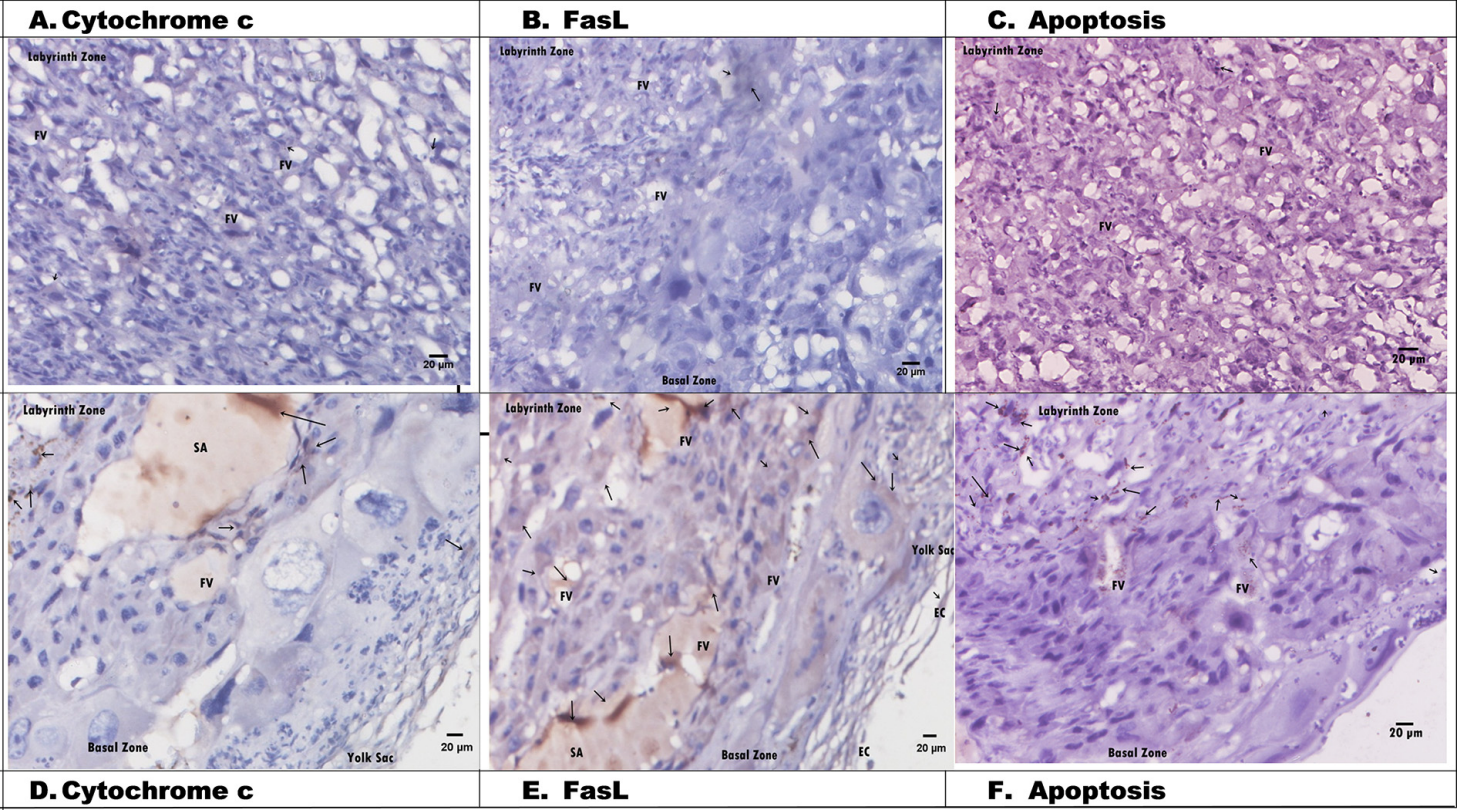
Histology of hypertension rats placental given *Extra virgin olive oil* (EVOO) (T1) A. Cytochrome *c* (20 µm), B. FasL (20 µm), C. Apoptosis (20 µm). D. Cytochrome *c* (20 µm), E. FasL (20 µm), F. Apoptosis (20 µm). EC: Epithelial Cells, FV: Fetal vessels. SA: Spiral artery.

Fig. 4 shows the histological changes in placental tissue apoptosis via cytochrome *c* and FasL after EVOO administration. FasL expression increased in the labyrinth zone and in the area near the FV (Fig. 4E). FasL could induce apoptosis, thereby preventing excessive chorion trophoblast cells from invading the uterine epithelium. Apoptosis in the T1 group was reduced compared with that in the hypertension group. Based on the placental histology of the T1 group, apoptosis via cytochrome *c* and FasL expression decreased due to EVOO administration.

### 3.6 Histological changes in placental tissue apoptosis via cytochrome *c* and FasL in hypertensive pregnant rats after administration of nanoherbal ZA

**Fig. 5:**
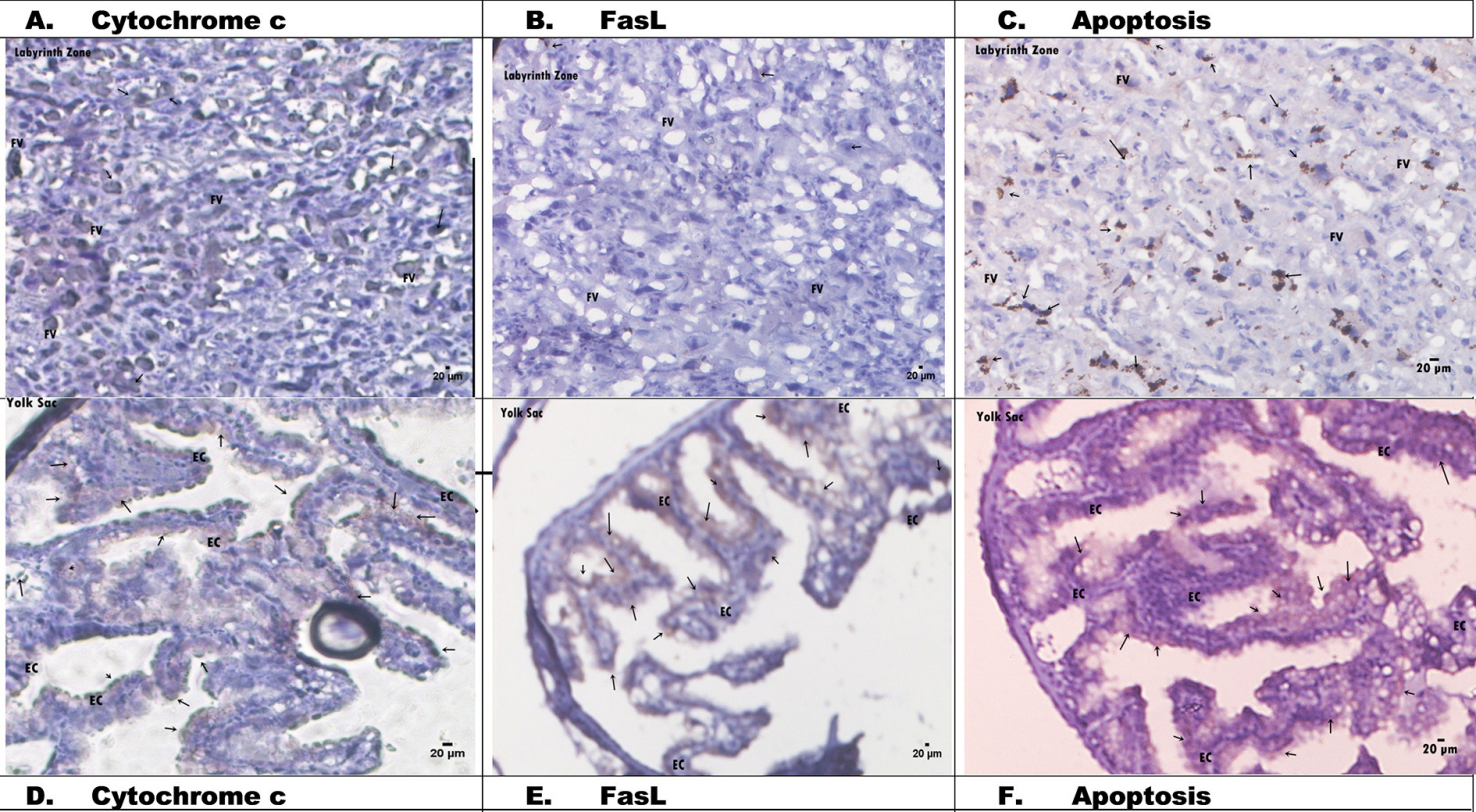
Histology of hypertension rats placental given nanoherbal ZA (T2) A. Cytochrome *c* (20 µm), B. FasL (20 µm), C. Apoptosis (20 µm). D. Cytochrome *c* (20 µm), E. FasL (20 µm), F. Apoptosis (20 µm). EC: Epithelial Cells, FV: Fetal vessels.

Fig. 5 shows cytochrome *c* expression in the labyrinth and basal zones after the administration of nanoherbal of ZA (Fig. 5A and D). Cytochrome *c* expression was also distributed along with the EC in the yolk sac. These tissues integrated with one another to form the interface between the foetal and maternal placenta. In this group, the apoptotic distribution was greater than those of FasL and cytochrome *c*. The number of apoptotic cells underwent a slight change. The type of apoptotic cell shown in this group was trophoblast cells in the foetal compartment in the maternal placental compartment. Based on the placental histology of the T2 group, apoptosis via cytochrome *c* and FasL expression decreased after administration of nanoherbal of ZA*.*

### 3.7 Histological changes in placental tissue apoptosis via cytochrome *c* and FasL in hypertensive pregnant rats after EVOO and nanoherbal ZA

**Fig. 6:**
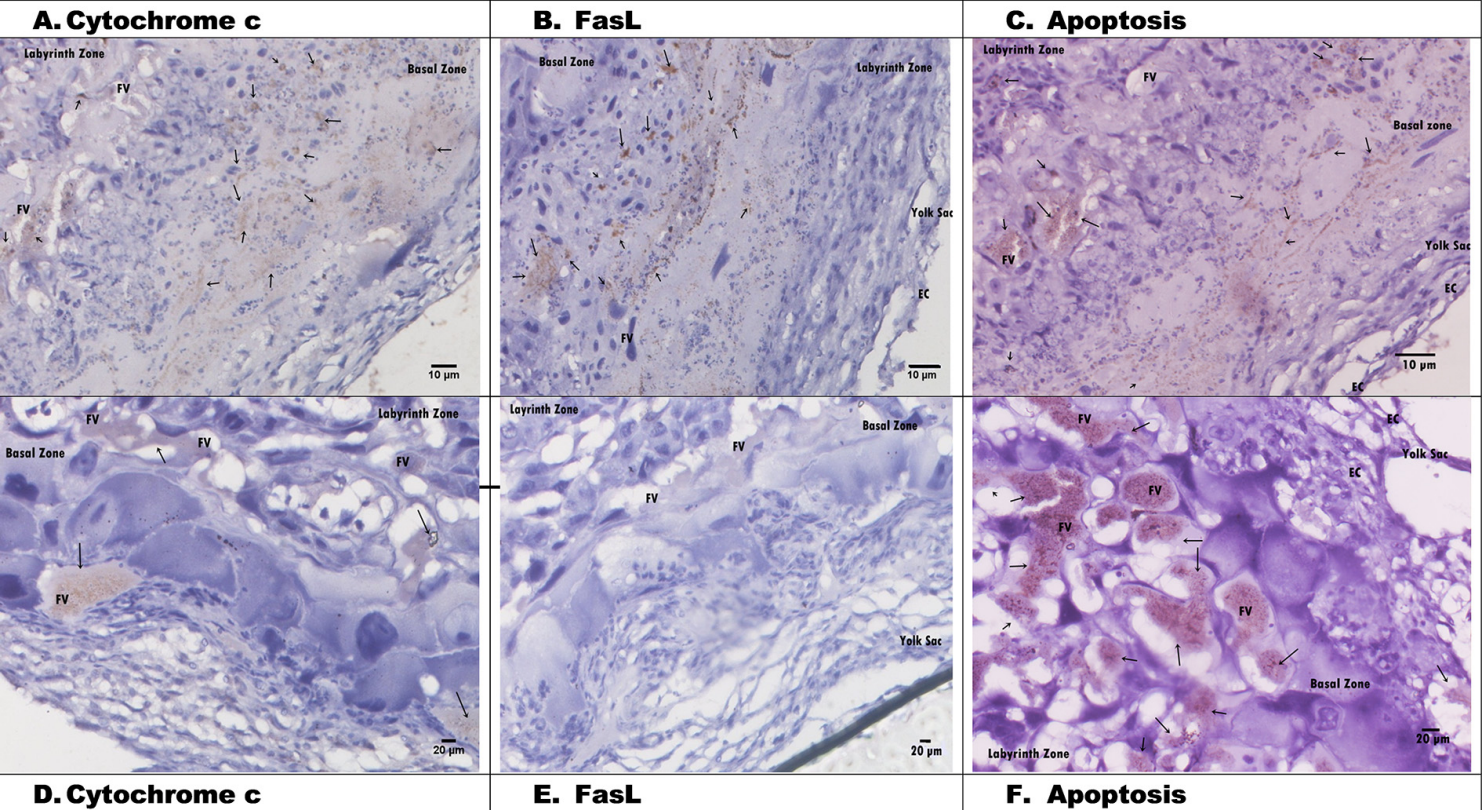
Histology of hypertension rats placental given EVOO and nanoherbal ZA (T3). A. Cytochrome *c* (20 µm), B. FasL (20 µm), C. Apoptosis (20 µm). D. Cytochrome *c* (20 µm), E. FasL (20 µm), F. Apoptosis (20 µm). EC: Epithelial Cells, FV: Fetal vessels.

Fig. 6 shows histological changes in apoptosis via cytochrome *c* and FasL expression by the combination of these two drugs. FasL was clearly visible in the labyrinth zone and was slightly reduced in the yolk sac (Fig. 6B and E). Trophoblast cell apoptosis after the administration of these two drug combinations was observed in the cavity around the basal zone and was decreased in the yolk sac (Fig. 6C and F). Based on the placental histology of the T3 group, apoptosis via cytochrome *c* and FasL expression was decreased compared with that of the hypertension group.

### 3.8 Statistical analysis of the positive indices FasL, cytochrome *c*, and apoptotic cells

A significant difference was found in FasL expression (P < 0.0001). Significant differences were also found in the T1, T2, and T3 groups (P < 0.001) compared with the hypertension (C+) group. Based on Table 2, FasL expression influenced placental tissue histology after the administration of EVOO and nanoherbal of ZA

**Table 2:** The Positive index of FasL expression on placental histology.

Groups	Mean Rank	Kruskal-Wallis	Mann-Whitney				
			K−	K+	P1	P2	P3
C-	20.33	0.000		0.007**	0.020*	0.032*	0.025*
C+	32.10				0.004**	0.035*	0.001**
T1	19.11					0.025**	0.026*
T2	21.83						0.002**
T3	10.29						

C-: untreated pregnant rats, C+: Hypertension rats, T1; Hypertension rats + EVOO, T2: Hypertension rats + andaliman, T3: Hypertension rats + EVOO + andaliman. (*P < 0.05, **P < 0.01, ***P < 0.001, ^ns^ = P > 0.05/ Not significant).

Table 3 shows that cytochrome *c* expression affects the placental histology of hypertension after the administration of EVOO and nanoherbal of ZA (P < 0.0001). Strong expression was found in the T1 and T3 groups, which decreased the expression of cytochrome *c*.

**Table 3:** The Positive index of cytochrome *c* expression on placental histology.

Groups	Mean Rank	Kruskal-Wallis	Mann-Whitney				
			K−	K+	P1	P2	P3
C-	16.70	0.000		0.006**	0.056 ^ns^	0.004**	0.020*
C+	32.10				0.002**	0.042*	0.004**
T1	19.11					0.045*	0.040*
T2	21.83						0.005**
T3	10.29						

C-: untreated pregnant rats, C+: Hypertension rats, T1;Hypertension rats + EVOO, T2: Hypertension rats + andaliman, T3: Hypertension rats + EVOO + andaliman. (*P < 0.05, **P < 0.01, ***P < 0.001, ^ns^ = P > 0.05/ Not significant).

Table 4 shows the apoptotic values in each treatment group. The highest apoptosis rate was found in the C+ group, and the lowest apoptosis rate was found in the T1 group. A significant difference in the expression of apoptosis was noted with the *Kruskal-Wallis* test (F = 0.000 or P < 0.001). The *Mann-Whitney* test showed that there were significant differences in each treatment.

**Table 4:** The Positive index of apoptotic cells on placental histology.

Groups	Mean Rank	Kruskal-Wallis	Mann-Whitney				
			K−	K+	P1	P2	P3
C−	16.15	0.000		0.005**	0.035*	0.0028**	0.458 ^ns^
C+	39.22				0,002**	0.040*	0.002**
T1	14.22					0.035*	0.466 ^ns^
T2	22.90						0.02*
T3	15.77						

C-: untreated pregnant rats, C+: Hypertension rats, T1; Hypertension rats + EVOO, T2: Hypertension rats + andaliman, T3: Hypertension rats + EVOO + andaliman. (*P < 0.05, **P < 0.01, ***P < 0.001, ^ns^ = P > 0.05/ Not significant).

## 4 Discussion

The injection of 6% NaCl can increase systolic and diastolic blood pressure. These results are also the same as those of previous studies (Situmorang et al., 2019a, Ilyas et al., 2018). Blood pressure, MDA, and HSP-70 can be used as markers of preeclampsia or hypertension, with increased MDA levels and decreased HSP70 levels in preeclampsia. Administration of EVOO and nanoherbal of ZA can reduce blood pressure levels of MDA and increase levels of HSP70. Therefore, this herb can be developed as a hypertension drug.

The present study provided information on cytochrome *c* and FasL expression in the context of hypertension, which is a pregnancy disorder associated with important adverse outcomes for the mother and the foetus. In the present study, we used immunohistochemical techniques to analyse cytochrome *c* and FasL and TUNEL assay techniques for apoptosis. Hypertension could affect the number of foetal deaths (Fig. 1). The current research broadened our understanding of mother–foetal interactions. Hypertension could affect the weight of the foetus or the incidence of premature birth, which was related to maternal morbidity and long-term mortality (Riise et al., 2017). There were no significant (P > 0.05) effects on placental weight or the number of foetuses after EVOO and nanoherbal ZA administration, but these effects could affect foetal weight (P < 0.05) (Situmorang et al., 2019a, Situmorang et al., 2019b). The number of foetuses in hypertensive pregnant rats could indicate delayed implantation, impaired preimplantation, release of the zona pellucida, and delays in the blastocyst stage or in cell division (Situmorang et al., 2019a). Hypertension in pregnant rats affected the number of stillborn rat foetuses, and nano-herbal ZA administration affected the number of live-born rat foetuses.

Cytochrome *c* plays a role in apoptotic placental hypertension and PE through the mitochondrial or intrinsic pathway. In the C+ group (Fig. 3A and D), the highest cytochrome *c* expression was increased compared with the other groups (Figs. 3–6; parts of cytochrome *c*). The initial event in the initiation of apoptosis in the intrinsic pathway was the opening of the pore transition and swelling of mitochondrial permeability. The mitochondrial matrix ruptures, and the intermembrane of mitochondrial space proteins (cytochrome *c*) enters the cytoplasm through permeabilization (Holland et al., 2017, Sesso et al., 2012). Mitochondria play a key role in the activation of cytochrome *c* because they release various proapoptotic proteins from the intermembrane space into the cytosol. In placental histology, cytochrome *c* cleaves pro-caspase-9 and produces an active enzyme (Holland et al., 2017). Oxidative stress marked by increased cytochrome *c* expression indicated that ROS were increased and led to oxidative stress (Burton et al., 2017, Li et al., 2011).

In hypertension and PE, T cells express small amounts of Fas and FasL to reduce apoptosis. Moreover, such cells can increase the destruction of cytotrophoblasts (Yang et al., 2019). The reduced number of cytotrophoblasts that were invaded cannot sufficiently modify the SA (Stenqvist et al., 2013, Wong et al., 2019). Based on placental histology, FasL expression was lower than cytochrome *c* expression. Moreover, FasL expression on the surface was lower in T cell PE than in normal pregnancies (Chen et al., 2019). Decreased FasL expression was observed with the administration of EVOO (Fig. 4B and D) and nanoherbal of ZA (Fig. 5B and D) compared with the combination of these two drugs, which caused an increase in FasL expression (Fig. 6B and D). Decreased FasL levels in villous trophoblasts in the preeclamptic placenta caused an increase in T lymphocyte infiltration in the decidua basalis, which resulted in increased apoptosis in villous trophoblasts (Luckerath et al., 2011). The FasL transmembrane also has the potential to act as a receptor because tail signalling is rich in complete intracellular proline (Storniolo et al., 2017).

Apoptosis in the pregnant placenta provides a local mechanism for maternal immunotolerance in the foetus and is mediated by the Fas-FasL pathway. However, the absence of Fas expression in the epithelium of the caruncular crypts indicated that the placentome was not activated by the Fas/FasL pathway (Jiangfenget al., 2011). Fas/FasL-mediated apoptosis is required for the normal release of apoptotic signal dysfunction and foetal membranes, including decreased expression and maintenance of FAS from cellular FLICElike inhibiting protein, which causes foetal membranes in cattle (Merkis et al., 2010). FasL given only nanoherbal of ZA and the combination with EVOO could increase apoptosis in the intrinsic pathway, as proven by high cytochrome *c* expression.

An imbalance of the apoptotic pathway throughout pregnancy causes dangerous pathologies with placental dysfunction (Pierik et al., 2020). When normal trophoblastic invasion and modified spiral arteries (SAs) are disrupted, increased vascular resistance and decreased placental perfusion could result in a lack of nutrient and oxygen supply for normal foetal growth (Jiang et al., 2014, Krishna and Bhalerao, 2011). Therefore, supplements are needed for the prevention or treatment of hypertension in pregnant patients, and antioxidants are needed in pregnancy to prevent oxidative stress. EVOO from olives contains antioxidants, namely, vitamin E, hydroxytyrosol, and tyrosol, which have high antioxidant activity and can reduce MDA levels and increase activation of HSP-70 (Situmorang et al., 2019a). Nanoherbal of ZA has high antioxidant and had extremely small-sized at 0.440 μm and based on particle size analysis, it has an average diameter of distribution of 783.9 ± 173.4 nm (Situmorang et al., 2019a). The cytotoxicity activities in this plant can through cell cycle arrest, reducing cyclin D1, and increasing the expression of p53 (Satria et al., 2019). However, an n-hexane extract from ZA fruit given during the postimplantation period of 6–14 days had a negative effect on mice liver changed the colour and texture of the liver surface and increased damage to hepatocytes (Sabri et al, 2018).

## 5 Conclusion

In conclusion, we demonstrated that apoptosis via cytochrome *c* and FasL increased during hypertension pregnancy. The administration of nanoherbal of ZA reduced apoptosis via cytochrome *c* and FasL. But EVOO reduces apoptosis via cytochrome *c* and FasL more than nanoherbal of ZA. A significant difference was found in the combination of these two drugs. Nanoherbal may be a candidate drug in the treatment of hypertension because it reduces blood pressure, MDA levels, raises HSP70 levels, and reducing apoptotic cells.

## Declaration of Competing Interest

The authors declare that they have no known competing financial interests or personal relationships that could have appeared to influence the work reported in this paper.
